# Mini Aortic Valve Replacement Converted to Bentall Procedure Secondary to Chronic Type A Aortic Dissection

**DOI:** 10.7759/cureus.53838

**Published:** 2024-02-08

**Authors:** Sheryar Muhammad, Jeremiah Fowler, Bassel Kadi

**Affiliations:** 1 Anesthesiology and Critical Care, Corewell Health William Beaumont University Hospital, Royal Oak, USA

**Keywords:** incidental chronic type a dissection, surgical aortic valve replacement (savr), minimally invasive aortic valve replacement, cardiopulmonary bypass associated vasoplegia, prolonged aortic cross-clamp time, cardiopulmonary bypass circuit, mini thoracotomy

## Abstract

In our case, a 46-year-old female with severe aortic insufficiency presented for minimally invasive aortic valve replacement. The patient was taken to the operating room, where transesophageal echocardiography showed severe aortic regurgitation with prolapse of the non-coronary cusp. The patient was placed on a cardiopulmonary bypass machine with peripheral cannulation. The aorta was cross-clamped, and an aortotomy was made. Despite multiple attempts, the left main coronary ostium was not visible. A sternotomy was quickly performed, and a newly discovered chronic type A dissection, obscuring the left main coronary artery, was found. Seventeen minutes after the cross-clamp was placed, the left main was transected, and cardioplegia was delivered. The patient then underwent a Bentall procedure with an aortic valve and root replacement.

## Introduction

We present a case of severe aortic insufficiency with a newly intraoperatively discovered chronic type A dissection during mini-thoracotomy, which led to an aortic cross-clamp time of 17 minutes before the delivery of cardioplegia solution, resulting in myocardial stunning and prolonged cardiopulmonary bypass time.

Aortic cross-clamping decreases coronary perfusion and increases myocardial wall tension and oxygen demand. Subsequent myocardial stunning, leading to myocardial dysfunction, can occur after a brief period of ischemia, in as little as 15 minutes [1].

Prolonged cardiopulmonary bypass time leads to platelet dysfunction, coagulopathy, and vasoplegia, secondary to the activation of inflammatory pathways and nitric oxide synthase [2].

## Case presentation

A 46-year-old female with severe aortic insufficiency presented for minimally invasive aortic valve replacement. Her past medical history included hypertension, chronic diastolic heart failure, and chronic kidney disease. A pre-induction arterial line was placed, and the patient was taken to the operating room. Transesophageal echocardiography demonstrated normal biventricular systolic function (Video 1) and severe aortic regurgitation (Video 2). The ascending aorta appeared normal on transesophageal echocardiography (Video 3).

**Video 1:** Transesophageal echocardiogram demonstrating normal biventricular function.

**Video 2:** Transesophageal echocardiogram demonstrating severe aortic regurgitation.

**Video 3:** Transesophageal echocardiogram demonstrating a normal ascending aorta.

After a mini-thoracotomy, attempts to place a coronary sinus cardioplegia catheter were unsuccessful due to difficult anatomy. Cardiopulmonary bypass was initiated via peripheral femoral cannulation, the aorta was cross-clamped, and an aortotomy was performed. Multiple attempts to arrest the heart with antegrade cardioplegia were made, but the left main coronary artery ostium was not visible, and the surgeon noticed a chronic dissection flap obscuring the left main. The heart continued to beat with the cross-clamp in place while attempts were made to locate the left main ostium. A sternotomy was quickly performed after the patient started bleeding heavily, and a newly discovered chronic type A dissection was found, extending from the proximal aorta and obscuring the left main coronary artery. Seventeen minutes after the cross-clamp was applied, the left main was accessed, and cardioplegia was delivered. The patient underwent a Bentall procedure with an aortic valve and root replacement.

After the Bentall procedure and the unclamping of the aorta, transesophageal echocardiography demonstrated a return to baseline cardiac function with high-dose ionotropic and vasopressor support. Due to concerns for myocardial stunning secondary to delayed cardioplegia administration and prolonged cross-clamp on the beating heart, an attempt to place an Impella device lasted for 1.5 hours. However, the aortic valve could not be crossed with the guidewire. The patient was then weaned from bypass with high-dose pressors and inotropes. Despite these measures, she remained vasoplegic and coagulopathic. She was also treated with methylene blue for vasoplegia and Kcentra (prothrombin complex concentrate) for coagulopathy, and she received a massive transfusion. Approximately two hours after weaning from bypass, left ventricular contractility diminished due to persistent hypotension and ongoing myocardial hypoperfusion, and the patient stopped responding to pharmacologic support. She expired in the operating room.

## Discussion

Our patient presented to the emergency department with chest pain radiating to the back almost six months before the procedure; the patient had a computed tomographic angiography that was negative for aortic dissection. Although computed tomography angiography is the gold standard for diagnosing acute aortic dissection, sometimes there can be false negatives, although the rate of false negatives is extremely low. The sensitivity and specificity of computed tomography angiography to identify acute aortic dissection range from 94% to 100% and 77% to 100%, respectively [3].

A couple of days before the surgery, an attempt at coronary catheterization was made but aborted due to the high contrast load in the setting of chronic kidney disease stage 3. Consequently, the patient was taken to the operating room without knowledge of the coronary anatomic details. Additionally, the patient had not undergone coronary computed tomography before the procedure, which might have helped in detecting coronary anatomy anomalies, especially coronary ostial lesions. Multislice computed tomography coronary angiogram (CTCA) offers an alternative diagnostic modality with 95% sensitivity and 98% specificity in detecting coronary ostial lesions [4].

Our patient experienced a prolonged aortic cross-clamp time before the delivery of cardioplegia. Suspecting myocardial stunning, we made a plan for coming off cardiopulmonary bypass after a discussion with the surgeon. The plan involved attempting to come off the bypass machine using high-dose pressors, inotropes, intra-aortic balloon pump placement, and possible Impella placement if needed. After unclamping the aorta, the patient returned to baseline cardiac function on transesophageal echocardiography. We recommended weaning from bypass with intra-aortic balloon pump placement without the need for Impella placement. After discussing our recommendation with the surgeon, he felt that further support was necessary and opted for Impella placement.

However, the Impella placement proved difficult; the guidewire could not cross the aortic valve despite multiple attempts. Attempts at Impella placement were made via femoral, subclavian, and even through access via the aortic graft. Trying to place the Impella extended the cardiopulmonary bypass time by almost 90 more minutes, resulting in refractory vasoplegia and coagulopathy [2].

Due to the prolonged cardiopulmonary bypass time, our patient became significantly vasoplegic and coagulopathic. We treated our patient with the maximum dose of pressors and inotropes. We also tried methylene blue, which treats vasoplegia by competitively inhibiting nitric oxide. Additionally, the patient received a massive transfusion and Kcentra, which has been shown to reduce transfusion rates and blood loss after bypass [5].

## Conclusions

We learned from this case that a patient should never be brought to the operating room for cardiac surgery without knowing the coronary anatomic details, unless it is a life-threatening emergency. If coronary catheterization was aborted due to concerns of a high contrast requirement in the setting of chronic kidney disease, coronary computed tomography could have been useful in this case for evaluating coronary anatomy, especially for any coronary ostial disease.

Close communication between anesthesiologists and cardiac surgeons is paramount in cardiac anesthesia. We recommended weaning from bypass with intra-aortic balloon pump placement without Impella placement to minimize bypass time. In cardiac surgery operating rooms, every effort should be made to minimize cardiopulmonary bypass time as it can lead to irreversible and refractory vasoplegia and coagulopathy.
